# Appropriate Body Mass Index and Waist Circumference Cutoff for Overweight and Central Obesity among Adults in Cambodia

**DOI:** 10.1371/journal.pone.0077897

**Published:** 2013-10-21

**Authors:** Yom An, Siyan Yi, Annette Fitzpatrick, Vinay Gupta, Piseth Raingsey Prak, Sophal Oum, James P. LoGerfo

**Affiliations:** 1 School of Public Health, the National Institute of Public Health, Phnom Penh, Cambodia; 2 Institute of Biology, Medicine and Agriculture, Royal Academy of Cambodia, Phnom Penh, Cambodia; 3 Asia Health Policy Program, Walter H. Shorenstein Asia-Pacific Research Center, Freeman Spogli Institute for International Studies, Stanford University, Stanford, California, United States of America; 4 University of Washington Medical Center, Departments of Internal Medicine and Global Health, Seattle, Washington, United States of America; 5 Department of Preventive Medicine, Ministry of Health, Phnom Penh, Cambodia; 6 University of Health Sciences, Phnom Penh, Cambodia; Scientific Directorate, Bambino Hospital, Italy

## Abstract

**Background:**

Body mass index (BMI) and waist circumference (WC) are used in risk assessment for the development of non-communicable diseases (NCDs) worldwide. Within a Cambodian population, this study aimed to identify an appropriate BMI and WC cutoff to capture those individuals that are overweight and have an elevated risk of vascular disease.

**Methodology/Principal Findings:**

We used nationally representative cross-sectional data from the STEP survey conducted by the Department of Preventive Medicine, Ministry of Health, Cambodia in 2010. In total, 5,015 subjects between age 25 and 64 years were included in the analyses. Chi-square, Fisher’s Exact test and Student *t*-test, and multiple logistic regression were performed. Of total, 35.6% (*n = *1,786) were men, and 64.4% (*n = *3,229) were women. Mean age was 43.0 years (SD = 11.2 years) and 43.6 years (SD = 10.9 years) for men and women, respectively. Significant association of subjects with hypertension and hypercholesterolemia was found in those with BMI ≥23.0 kg/m^2^ and with WC >80.0 cm in both sexes. The Area Under the Curve (AUC) from Receiver Operating Characteristic curves was significantly greater in both sexes (all *p*-values <0.001) when BMI of 23.0 kg/m^2^ was used as the cutoff point for overweight compared to that using WHO BMI classification for overweight (BMI ≥25.0 kg/m^2^) for detecting the three cardiovascular risk factors. Similarly, AUC was also significantly higher in men (*p*-value <0.001) when using WC of 80.0 cm as the cutoff point for central obesity compared to that recommended by WHO (WC ≥94.0 cm in men).

**Conclusion:**

Lower cutoffs for BMI and WC should be used to identify of risks of hypertension, diabetes, and hypercholesterolemia for Cambodian aged between 25 and 64 years.

## Introduction

Body mass index (BMI) and waist circumference (WC) have been widely used to predict risks of cardiovascular disease including type II diabetes, hypertension, and dyslipidemia [1]–[4]. The World Health Organization (WHO) has classified BMI <18.5 kg/m^2^ as underweight, between 18.5–24.9 kg/m^2^ as normal weight, between 25.0–29.9 kg/m^2^ as overweight, and ≥30.0 kg/m^2^ as obese, WHO cut points for WC are classified as ≥94.0 cm for men and WC ≥80.0 cm for women to reflect central obesity [5]. These classifications are based mainly on studies from Western populations [5].

Increasingly, epidemiological and clinical studies have shown a significant association of BMI and WC at lower cutoff points with risks of metabolic disorders among Asian populations [4], [6]–[8]. A BMI threshold of ≥23.0 kg/m^2^ has been found to be associated with diabetes among Indian people [7]. In China, BMI of 22.5–24.0 kg/m^2^ was found to be associated with hypertension [1], [9], while this association was found at a lower BMI cutoff in Indonesia (from 21.5–22.5 kg/m^2^) and Vietnam (from 20.5–21.0 kg/m^2^) [9]. To define central obesity, measures of WC ≥90.0 cm for men and ≥80.0 cm for women are widely used for Asian people [10], [11]. In India, diabetes was found to be associated with those who had even lower WC (85.0 cm and 80.0 cm for men and women, respectively) [7]. In China, WC of 80.0 cm for both men and women was found as the threshold to confer risks of cardiovascular disease [6].

In Cambodia, BMI classification from the WHO [5] (BMI cutoff point of ≥25.0 kg/m^2^ for overweight) still has been used to identify people at greater risks of non-communicable diseases (NCDs). Men with WC of 85.0–94.0 cm and women with WC of 81.0–88.0 cm are classified as having moderate risk of NCDs. Using these classifications, a national STEP survey of risk factors for NCDs conducted by Department of Preventive Medicine, Ministry of Health, Cambodia in 2010 found that 10.5% of men and 16.3% of women were classified as overweight; and 11.8% of men and 16.9% of women were classified as having central obesity [12]. This prevalence is low compared to other neighboring countries such as Thailand [13] (overweight: 17.1%, obese: 23.8%) and Vietnam [14] (overweight: 27.5%, obese: 5.7%), where Asian BMI cutoffs is used.

The report of the Cambodian national STEP survey did not address the question of whether the usage of using lower BMI and WC classifications would be appropriate for estimating people at increased risks of NCDs in Cambodia. The question remains as to whether Cambodia should use the “Asian” BMI cutoffs of ≥23.0 kg/m^2^ for overweight and ≥28.0 kg/m^2^ for obesity and the “Asian” WC cutoffs of ≥90.0 cm for men and ≥80.0 cm for women for central obesity versus WHO recommendations. This lack of information needs to be answered in order to appropriately inform policy makers and those who are concerned with controlling NCDs in Cambodia. Therefore, the objectives of this study were to determine appropriate BMI and WC cutoff for overweight and central obesity and their associations with CVD risk factors for adults aged 25 to 64 years in Cambodia and to determine whether those cutoffs are more appropriate than the WHO cutoffs.

## Methods

### Ethics Statement

All participants were fully explained about the nature and possible consequences of the study. Privacy and anonymity of respondents were fully guaranteed. Respondents have right to quit from the research at any time without any explanation or reason. Verbal explanation was done with those who were illiterate. A written informed consent was obtained from all subjects prior to data collection. This study was approved by the National Ethics Committee for Health Research, Ministry of Health, Cambodia.

### Study Population

We used data from the 2010 STEP survey conducted by the Department of Preventive Medicine, Ministry of Health, Cambodia. It is a nationwide cross-sectional survey that was carried out from February to April 2010 using the WHO STEPwise approach to chronic disease risk factor surveillance methodology [15]. The survey was led by a research team from the University of Health Sciences with technical support from the WHO. A multistage-cluster sampling was used to randomly select participants. Communes were randomly selected as the primary sampling unit, followed by villages as the secondary sampling unit and households as elementary units. In total, 5,643 participants were randomly selected accounting for equivalent distribution of gender and age groups (10-years age groups). Of those, 5,433 individuals aged 25 to 64 years participated in the survey, a response rate of 96.3%. For these analyses, we excluded all subjects who had missing values in main variables such as blood pressure (*n = 118*), height (*n = 5*), fasting blood sugar (*n = 188*), and total cholesterol (*n = 19*). We also excluded 87 pregnant women and one subject with abnormal values in the main variables from the analysis. In total, 418 subjects were excluded resulting in a total of 5,015 persons analyzed here.

### Variables and Measurements

#### Physical and biological measurements

Blood pressure was measured three times on the left arm at sitting position using NISSEL digital blood pressure monitor (model DS-500) automatic digital blood pressure equipment. The 1^st^ measurement of blood pressure was taken after 15 mn rest and the 2^nd^ and 3^rd^ measurements were made after 3 mn interval. Hypertension was defined as systolic blood pressure or diastolic blood pressure, calculated from the means of the last two readings, of ≥140 mmHg and ≥90 mmHg, respectively. Participants who were currently on anti-hypertensive medication were also classified as hypertensive cases. Weight and height were measured using Linkfold electronic body scale, HCS-200-RT model, made by Shanghai Medical Instrument Co. Ltd with the capacity to measure weight up to 200 kg and height up to 210 cm with a precision of 100 grams and 0.5 cm for weight and height, respectively. Weight was measured in light indoor clothing and without footwear. BMI was calculated as the weight in kilograms divided by the square of the height in metre square. A tape with mm(s) precision made from linoleum was used to measure WC. It was measured at standing position at midpoint of the last palpable rib and the iliac crest.

A blood sample was drawn after subjects had fasted overnight. Fasting blood glucose and total cholesterol were measured by trained laboratory technicians using capillary drop of blood from participants’ finger. Accutrend Plus instruments were used for these measurements and Accutrend control glucose and cholesterol solutions were used to calibrate each instrument at least twice a week. Diabetes mellitus was defined as subjects who had fasting blood glucose of ≥126 mg/dl or those who were currently on medications for diabetes. Hypercholesterolemia was defined as those with total cholesterol of ≥190 mg/dl.

#### Socio-demographic characteristics and living behaviors

Measures from the STEPS survey included age (continuous), sex (male or female), residence (rural or urban), ethnicity (Khmer or other), education level (completed primary school, completed secondary school, and post high school), marital status (married, single, other), employment status (employed, unemployed), cigarette smoking status (yes or no), smokeless tobacco use status (yes or no), alcohol drinking status (yes or no), fruit and/or vegetable consumption ≥5 servings per day (yes or no) and using lard/suet as cooking oil (yes or no). These data were collected by trained interviewers using a questionnaire adapted from WHO STEPwise after translating into Khmer language by taking into consideration specific country characteristics. The interview took approximately 30 minutes.

### Statistical Snalyses

Data analyses were performed using STATA version 11.0. BMI was stratified as <18.5 kg/m^2^, 18.5–<23.0 kg/m^2^, 23.0–<27.5 kg/m^2^, and ≥27.5 kg/m^2^. This classification was adapted from WHO expert panel recommendation potential BMI categories for public health action in people of Asian ethnicity [11]. A BMI of 18.5–<23.0 kg/m^2^ was taken as reference. WC was also categorized in units of 10 cm for both sexes. WC of ≤70.0 cm and >90.0 cm were used as the lowest and highest unit, respectively, and WC of >70.0–80.0 cm was used as reference group.

Prevalence and means of socio-demographic characteristics and NCD risk factors of participants were calculated and differences were tested using Chi-square, Fisher’s Exact test for categorical variables and Student *t*-test for continuous variables. Associations with BMI and WC categories with hypertension, diabetes mellitus, and hypercholesterolemia were examined. Crude and adjusted odds ratio and 95% confidence interval (CI) were calculated using bivariate and multivariate logistic regression analysis, respectively. Based on previous literature [16]–[20], potential confounders were adjusted. In Model 1, we adjusted for age, sex, and residence. In model 2, we additionally adjusted for physical activities, cigarette smoking, alcohol drinking, fruit and/or vegetable consumption, and using lard/suet as cooking oil. Two-sided *p*-values of ≤0.05 were regarded as statistically significant. In order to test whether the lower cutoffs have at least as great a biologic plausibility for assessing risks as for the WHO cutoffs, we constructed receiver operating characteristic (ROC) curves for the ability of each of the cutoffs of BMI and WC to detect one or more components of the metabolic syndrome. Areas under the curve (AUC) were computed for the different cut-points of BMI and WC [21].

## Results

Socio-demographic characteristics of the study subjects are shown in Table 1. Of 5,015 subjects who were included in the analysis, 35.6% (*n = *1,786) were men, and 64.4% (*n = *3,229) were women. Mean age was 43.0 years (SD = 11.2 years) for men and 43.6 years (SD = 10.9 years) for women. Annual income for both sexes was not significantly different (USD 1,121± USD 1,545 for men vs. USD1,230± USD 2,358 for women). The distribution of men and women in urban (17.2% vs. 18.0%) and rural (82.8% vs. 82.0%) was also similar. Compared to women, men were significantly more likely to have completed high school education (18.7% vs. 7.7%), to be married (90.6% vs. 65.5%), to be employed (96.6% vs. 83.4%), to be current cigarette smokers (55.9% vs 6.4%), to be former daily smokers (38.0% vs. 2.9%), to be alcohol drinkers (89.4% vs. 56.4%), and to do regular physical activities (66.1% vs. 52.6%). However, women were more likely to be smokeless tobacco users than men (0.8% in men vs. 1.5% in women).

**Table 1:** Socio-demographic of participants.

	Men	Women	*p*-value
**Subject**, n (%)	1,786 (35.6)	3,229 (64.4)	
**Age in year** (mean ± sd)	43.0±11.2	43.6±10.9	0.96
**Annual income in USD** (mean ± sd)	1,121.2±1,545.5	1,230.8±2,358.0	0.95
**Residence**, n (%)			
Urban	307 (17.2)	581 (18.0)	0.47
Rural	1,479 (82.8)	2,648 (82.0)	
**Education level**, n (%)			
Completed Primary School	1,418 (79.4)	2,969 (91.9)	<0.001
Completed High School	334 (18.7)	250 (7.7)	
>High School	33 (1.8)	10 (0.3)	
**Ethnicity**, n (%)			
Khmer	1,761 (99.8)	3,190 (99.4)	0.04
Other	3 (0.2)	18 (0.6)	
**Marital status**, n (%)			
Single	66 (3.7)	188 (5.8)	<0.001
Married	1,619 (90.6)	2,116 (65.5)	
Other*	101 (5.7)	924 (28.6)	
**Employment status**, n (%)			
Employed	1,726 (96.6)	2,694 (83.4)	<0.001
Unemployed	60 (3.4)	535 (16.6)	
**Current cigarette smokers**, n (%)			
Yes	999 (55.9)	207 (6.4)	<0.001
No	787 (44.1)	3,022 (93.6)	
**Former daily smokers**, n (%)			
Yes	333 (38.0)	89 (2.9)	<0.001
No	543 (62.0)	2,973 (97.1)	
**Current smokeless tobacco users** **, n (%)			
Yes	46 (2.6)	622 (19.3)	<0.001
No	1,740 (97.4)	2,607 (80.7)	
**Former daily smokeless tobacco users**, n (%)			
Yes	15 (0.8)	41 (1.5)	0.05
No	1,747 (99.1)	2,622 (98.5)	
**Ever drink alcohol**, n (%)			
Yes	1,597 (89.4)	1,821 (56.4)	<0.001
No	189 (10.6)	1,408 (43.6)	
**Physical activity** ***, n (%)			
Yes	1,180 (66.1)	1,700 (52.6)	<0.001
No	606 (33.9)	1,529 (47.3)	

* Separated, Divorced, Widowed.

** Snuff, chewing tobacco, betel.

*** Physical activity is defined as vigorous-intensity aerobic physical activity for at least 75 mm throughout the week.

Table 2 shows the prevalence of risk factors of NCD across different categories of BMI and WC. The proportion of participants in BMI category of 18.5–<23.0 kg/m^2^, 23.0–<27.5 kg/m^2^, and ≥27.5 kg/m^2^ was 53.9%, 25.5%, and 6.9%, respectively. Of total, 20.0% of participants had WC between >80.0–90.0 cm, and only 6.9% of them had WC of >90.0 cm. In general, women, older people, urban residents, those who did not do regular physical activities, non-smokers, and those who did not use lard or suet as cooking oil were significantly more likely to have BMI ≥23.0 kg/m^2^ (*p*-values <0.001) in relation to their comparison groups. Similarly, women, older people, urban residents, and those who did not do physical activities, non-smokers, and those who did not use lard or suet as cooking oil were significantly more likely to have WC >80.0–90.0 cm (*p*-values <0.01) in relation to their comparison groups.

**Table 2:** Prevalence of risk factors of non-communicable diseases (NCDs) across different stratifications of body mass index (BMI) and waist circumference (WC).

	BMI Category (kg/m^2^)					WC Category (cm)				
	<18.5	18.5–<23	23–<27.5	≥27.5	*p*-value	≤70	>70–80	>80–90	>90	*p*-Value
**Subject distribution**	683 (13.6)	2,705 (53.9)	1,279 (25.5)	348 (6.9)		1,514 (30.2)	2,080 (41.5)	1,003 (20.0)	418 (6.9)	
**Gender**										
Male	179 (10.0)	1,123 (62.9)	409 (22.9)	75 (4.2)	<0.001	437 (24.5)	896 (50.2)	330 (18.5)	123 (6.9)	<0.001
Female	504 (15.6)	1,582 (49.0)	870 (26.9)	273 (8.4)		1,077 (33.3)	1,184 (36.7)	673 (20.8)	295 (9.1)	
**Age**										
25–34	150 (11.8)	822 (64.8)	249 (19.6)	48 (3.8)	<0.001	519 (40.9)	561 (44.2)	156 (12.3)	33 (2.6)	<0.001
35–44	151 (10.8)	738 (52.9)	399 (28.6)	108 (7.7)		386 (27.6)	595 (42.6)	296 (21.2)	119 (8.5)	
45–54	184 (13.4)	670 (48.7)	408 (29.6)	115 (8.3)		325 (23.6)	562 (40.8)	344 (25.0)	146 (10.6)	
55–64	198 (20.3)	475 (48.8)	223 (22.9)	77 (7.9)		284 (29.2)	362 (37.2)	207 (21.3)	120 (12.3)	
**Residence**										
Urban	68 (7.7)	354 (39.9)	333 (37.5)	133 (15.0)	<0.001	155 (17.4)	296 (33.3)	261 (29.4)	176 (19.8)	<0.001
Rural	615 (14.9)	2,351 (57.0)	946 (22.9)	215 (5.2)		1,359 (32.9)	1,784 (43.2)	742 (18.0)	242 (5.9)	
**Physical activities**										
Yes	678 (13.1)	1,653 (57.4)	675 (23.4)	174 (6.0)	<0.001	916 (31.8)	1,253 (43.5)	516 (17.9)	195 (6.8)	<0.001
No	305 (14.3)	1,052 (49.3)	604 (28.3)	174 (8.1)		598 (28.0)	827 (38.4)	487 (22.8)	223 (10.4)	
**Cigarette smoking**										
Yes	164 (13.6)	767 (63.6)	232 (19.2)	43 (3.6)	<0.001	340 (28.2)	598 (49.6)	195 (16.2)	73 (6.1)	<0.001
No	519 (13.6)	1,938 (50.9)	1,047 (27.5)	305 (8.0)		1,174 (30.8)	1,482 (38.9)	808 (21.2)	345 (9.1)	
**Alcohol drinking** 1										
Yes	227 (9.8)	1,337 (58.0)	600 (26.0)	141 (6.2)	0.05	608 (25.4)	1,063 (46.1)	470 (20.4)	164 (7.1)	<0.001
No	67 (13.2)	271 (53.2)	132 (25.9)	39 (7.7)		161 (31.6)	201 (39.5)	98 (19.2)	49 (9.6)	
**Fruit/veg. consumption** **≥5 serving/day**										
Yes	236 (13.4)	963 (54.6)	437 (24.8)	128 (7.3)	0.7	524 (29.7)	729 (41.3)	345 (19.6)	166 (9.4)	0.5
No	441 (13.8)	1,716 (53.6)	830 (25.9)	214 (6.7)		974 (30.4)	1,333 (41.6)	646 (20.2)	248 (7.7)	
**Using lard/suet as** **cooking oil**										
Yes	118 (16.0)	425 (57.6)	165 (22.4)	30 (4.1)	<0.001	255 (34.5)	314 (42.5)	130 (17.6)	39 (5.3)	0.003
No	564 (13.2)	2,280 (53.3)	1,114 (26.0)	318 (7.4)		1,258 (29.4)	1,766 (41.3)	873 (20.4)	379 (8.9)	

1 Drink alcohol within the last 30 day.

Table 3 shows non-adjusted and adjusted odds ratios for the association between hypertension, diabetes mellitus, and hypercholesterolemia and levels of BMI in men and women separately in three statistical models. BMI category of 18.5–<23.0 kg/m^2^ was used as reference group. In men, compared to the reference category, those with BMI of 23.0–<27.5 kg/m^2^ were significantly more likely to have hypertension in all models (OR = 2.4, 95% CI = 1.8–3.2; OR = 2.1, 95% CI = 1.6–2.9; OR = 2.4, 95% CI = 1.7–3.3 in model 0, model 1, and model 2, respectively). The increased risk of hypertension was also found in men in BMI category of ≥27.5 kg/m^2^ compared to those in reference category in all models (OR = 4.9, 95% CI = 3.0–8.1; OR = 3.6, 95% CI = 2.1–6.2; OR = 3.3, 95% CI = 1.8–6.1 in model 0, model 1, and model 2, respectively). Similarly, men in BMI category of 23.0–<27.5 kg/m^2^ were significantly more likely to have hypercholesterolemia compared to those in reference category in all three models (OR = 3.0, 95% CI = 2.3–4.0; OR = 2.7, 95% CI = 2.0–3.6; OR = 2.9, 95% CI = 2.1–4.0 in model 0, model 1, and model 2, respectively). The increased risk of hypercholesterolemia among men in BMI category of ≥27.5 kg/m^2^ compared to men in reference category was also statistically significant in all models (OR = 6.7, 95% CI = 4.1–10.8; OR = 5.1, 95% CI = 3.1–8.4; OR = 4.9, 95% CI = 2.8–8.6 in model 0, model 1, and model 2, respectively). Compared to men in reference category, men in BMI category of 23.0–<27.5 kg/m^2^ were significantly more likely to be diabetic in all models (OR = 4.0, 95% CI = 1.9–8.5; OR = 3.9, 95% CI = 1.4–6.4; OR = 3.4, 95% CI = 1.3–9.3 in model 0, model 1, and model 2, respectively). However, the increased risk of diabetes mellitus among men in BMI category of ≥27.5 kg/m^2^ compared to those in reference category was statistically significant only in the unadjusted model (OR = 5.2, 95% CI = 1.6–16.6).

**Table 3:** Odd ratio (OR) of hypertension, diabetes mellitus and hypercholesterolemia across body mass index (BMI) category.

	BMI Category (kg/m^2^)					
	<18.5 OR(95% CI)		18.5–<23Reference	23–<27.5 OR(95% CI)		≥27.5 OR(95% CI)
**MEN**						
** Hypertension**						
Model 0		0.8 (0.4–1.3)	1.0		2.4^c^ (1.8–3.2)	4.9^c^ (3.0–8.1)
Model 1		0.6 (0.3–1.0)	1.0		2.1^c^ (1.6–2.9)	3.6^c^ (2.1–6.2)
Model 2		0.7 (0.4–1.4)	1.0		2.4^c^ (1.7–3.3)	3.3^c^(1.8–6.1)
** Diabetes mellitus**						
Model 0		1.0 (0.2–4.7)	1.0		4.0^c^ (1.9–8.5)	5.2^b^ (1.6–16.6)
Model 1		0.9 (0.2–3.6)	1.0		3.0^b^ (1.4–6.4)	2.5 (1.7–8.4)
Model 2		2.1 (0.4–10.4)	1.0		3.4^a^ (1.3–9.3)	2.6 (0.5–13.6)
** Hypercholesterolemia**						
Model 0		0.8 (0.5–1.3)	1.0		3.0^c^ (2.3–4.0)	6.7^c^ (4.1–10.8)
Model 1		0.7 (0.4–1.2)	1.0		2.7^c^ (2.0–3.6)	5.1^c^ (3.1–8.4)
Model 2		0.7 (0.3–1.4)	1.0		2.9^c^ (2.1–4.0)	4.9^c^ (2.8–8.6)
**WOMEN**						
** Hypertension**						
Model 0		0.8 (0.6–1.2)	1.0		2.4^c^ (1.9–3.1)	3.9^c^ (2.8–5.3)
Model 1		0.6^b^ (0.4–1.0)	1.0		2.3^c^ (1.8–2.9)	3.3^c^ (2.3–4.6)
Model 2		0.8 (0.4–1.5)	1.0		2.0^c^ (1.3–3.0)	2.9^c^(1.7–5.1)
** Diabetes mellitus**						
Model 0		0.3 (0.1–1.0)	1.0		2.0^b^ (1.2–3.1)	2.7^c^ (1.5–5.0)
Model 1		0.3^a^ (0.1–0.8)	1.0		1.6^a^ (1.0–2.6)	1.9^a^ (1.0–3.6)
Model 2		0.8 (0.2–4.2)	1.0		1.4 (0.5–4.0)	3.0 (0.9–9.2)
** Hypercholesterolemia**						
Model 0		0.8 (0.6–1.1)	1.0		2.1^c^ (1.2–2.6)	2.9^c^ (2.2–3.7)
Model 1		0.8^a^ (0.6–1.0)	1.0		1.9^c^ (1.6–2.3)	2.3^c^ (1.7–3.0)
Model 2		1.1 (0.7–1.8)	1.0		2.1^c^ (1.5–2.9)	2.0^b^ (1.2–3.2)
**BOTH SEXES**						
** Hypertension**						
Model 0	0.8 (0.6–1.0)		1.0	2.3^c^ (1.9–2.8)		3.9^c^ (3.0–5.0)
Model 1	0.6^b^ (0.4–0.8)		1.0	2.2^c^ (1.8–2.7)		3.3^c^ (2.5–4.4)
Model 2	0.8 (0.5–1.2)		1.0	2.2^c^ (1.7–2.9)		3.1^c^(2.1–4.6)
** Diabetes mellitus**						
Model 0	0.5 (0.2–1.2)		1.0	2.5^c^ (1.7–3.7)		3.4^c^ (2.0–5.9)
Model 1	0.4^a^ (0.2–0.9)		1.0	1.9^b^ (1.3–2.9)		2.0^a^ (1.1–3.6)
Model 2	1.3 (0.4–3.9)		1.0	2.2^a^ (1.3–2.9)		3.0^a^ (1.2–7.7)
** Hypercholesterolemia**						
Model 0	0.9 (0.7–1.1)		1.0	2.5^c^ (2.1–2.9)		3.7^c^ (2.9–4.7)
Model 1	0.8^a^ (0.6–1.0)		1.0	2.1^c^ (1.8–2.5)		2.7^c^ (2.1–3.5)
Model 2	1.0 (0.7–1.4)		1.0	2.4^c^ (1.9–3.0)		2.8^c^ (2.0–4.0)

a *p*-value <0.05;

b *p*-value <0.01;

c *p*-value <0.001.

Model 0: Non adjusted.

Model 1: Adjusted for age, sex, residence,

Model 2: Adjusted for age, sex, residence, physical activities, cigarette smoking, alcohol drinking, fruit and/or vegetable consumption, using lard/suet.

In women, compared to those in the reference category, women in BMI category of 23.0– <27.5 kg/m^2^ were significantly more likely to have hypertension in all models (OR = 2.4, 95% CI = 1.9–3.1; OR = 2.3, 95% CI = 1.8–2.9; OR = 2.0, 95% CI = 1.3–3.0 in model 0, model 1, and model 2, respectively). As expected, the increased risk of hypertension was found in the comparison of women in BMI category of ≥27.5 kg/m^2^ and those in reference category (OR = 3.9, 95% CI = 2.8–5.3; OR = 3.3, 95% CI = 2.3–4.6; OR = 2.9, 95% CI = 1.7–5.1 in model 0, model 1, and model 2, respectively). Similarly, women in BMI category of 23.0–<27.5 kg/m^2^ were significantly more likely to have hypercholesterolemia in all models (OR = 2.1, 95% CI = 1.2–2.6; OR = 1.9, 95% CI = 1.6–2.3; OR = 2.1, 95% CI = 1.5–2.9 in model 0, model 1, and model 2, respectively) compared to those in reference category. The increased risk of hypercholesterolemia also remained statistically significant in comparisons of women in BMI category of ≥27.5 kg/m^2^ with those in reference category in all models (OR = 2.9, 95% CI = 2.2–3.7; OR = 2.3, 95% CI = 1.7–3.0; OR = 2.0, 95% CI = 1.2–3.2 in model 0, model 1, model 2, respectively). However, women in BMI category of 23.0–<27.5 kg/m^2^ were significantly more likely to have diabetes mellitus only in the unadjusted model (OR = 2.0, 95% CI = 1.2–3.1) and model 1 (OR = 1.9, 95% CI = 1.6–2.3) in comparison with reference group. The statistical association disappeared after additional adjustment in model 2.

**Figure 1:**
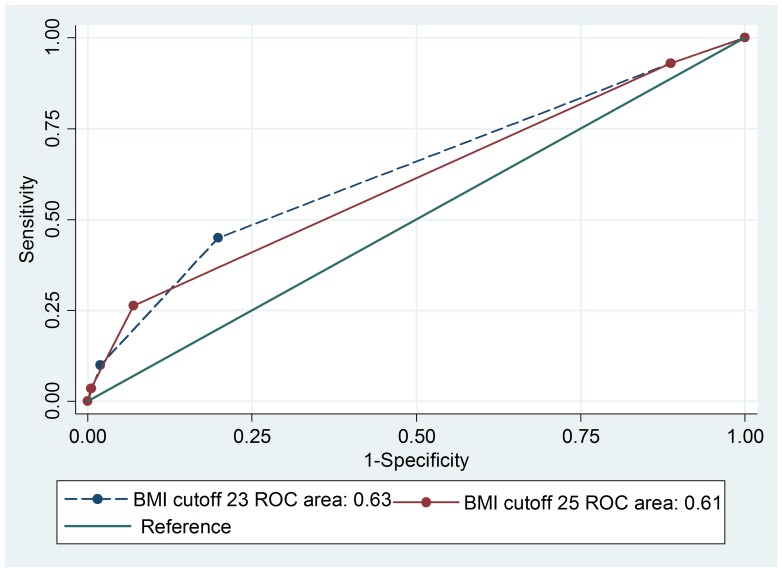
ROC curve comparing two BMI categories for predicting hypertension, diabetes mellitus and hypercholesterolemia in men.

**Figure 2:**
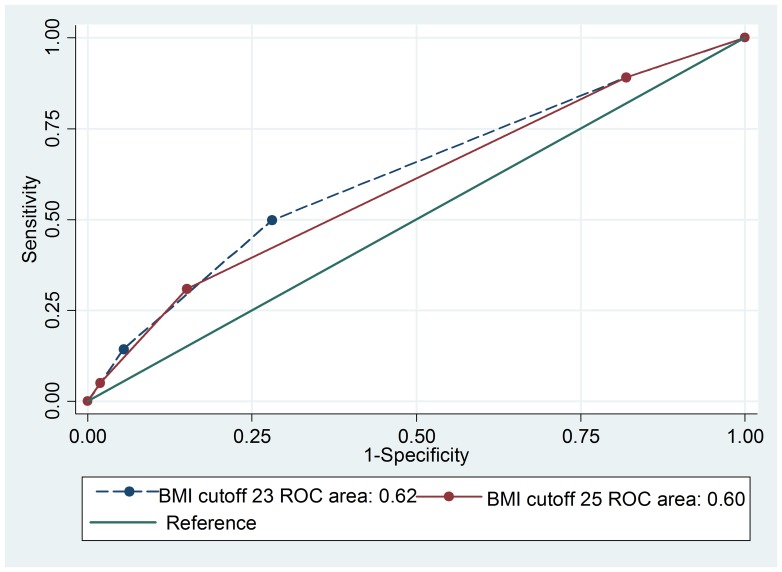
ROC curve comparing two BMI categories for predicting hypertension, diabetes mellitus and hypercholesterolemia in women.

In men, the ROC analysis showed greater area under curve (AUC) for a BMI cut-off of 23.0 kg/m^2^ (0.63) than for BMI cut-off of 25.0 kg/m^2^ (0.61) with *p*-value <0.001 (Figure 1). The sensitivity for detecting one of the metabolic syndrome components (diabetes, hypertension, or hypercholesterolemia) using a BMI cut-off of 23.0 kg/m^2^ was 45.0% compared to only 26.0% if a BMI cutoff of 25.0 kg/m^2^ was used to reflect overweight status. The corresponding specificities were 80.0% and 93.0% when BMI cutoff of 23.0 kg/m^2^ and 25.0 kg/m^2^ were used, respectively. Similarly, in women, the AUC from the ROC analysis was significantly greater for a BMI cut-off of 23.0 kg/m^2^ (0.62) than for BMI cut-off of 25.0 kg/m^2^ (0.60) with *p*-value <0.001 (Figure 2). The sensitivity for detecting one of the metabolic syndrome components using BMI cut-off of 23.0 kg/m^2^ was 49.0% compared to only 30.0% if BMI cutoff of 25.0 kg/m^2^ was used for overweight. The corresponding specificities were 71.0% and 84.0% when BMI cutoff of 23.0 kg/m^2^ and 25.0 kg/m^2^ were used, respectively.

Non-adjusted and adjusted odds ratios of the association of hypertension, diabetes mellitus, and hypercholesterolemia across stratifications of WC in men and women are shown in Table 4. WC of >70.0–80.0 cm was used as the reference category. In general, men and women with WC >80.0 cm were at significantly increased risk of hypertension, diabetes mellitus, and hypercholesterolemia (all *p*-value <0.05) except diabetes mellitus in Model 2 (*p*-value >0.05).

**Table 4:** Odd ratio (OR) of diabetes mellitus, hypertension and hypercholesterolemia across waist circumference (WC) category.

	WC Category (cm)			
	≤70 OR (95% CI)	>70–80 Reference	>80–90 OR (95% CI)	>90 OR (95% CI)
**MEN**				
** Hypertension**				
Model 0	0.6^b^ (0.4–0.9)	1.0	2.2^c^ (1.6–3.0)	5.2^c^ (3.4–7.8)
Model 1	0.6^b^ (0.5–0.9)	1.0	1.9^c^ (1.7–2.6)	3.9^c^ (2.5–6.1)
Model 2	0.7 (0.4–1.1)	1.0	1.9^b^ (1.3–2.8)	4.2^c^ (2.6–6.9)
** Diabetes mellitus**				
Model 0	0.4 (0.1–1.9)	1.0	4.8^c^ (2.2–10.6)	4.5^b^ (1.6–12.7)
Model 1	0.4 (0.1–1.9)	1.0	3.6^b^ (1.6–8.0)	2.2 (0.7–6.5)
Model 2	0.7(0.1–3.6)	1.0	3.3^a^ (1.2–9.1)	1.8 (0.4–7.7)
** Hypercholesterolemia**				
Model 0	0.8 (0.6–1.2)	1.0	3.5^c^ (2.6–4.8)	6.5^c^ (4.3–9.8)
Model 1	0.8 (0.6–1.2)	1.0	3.2^c^ (2.4–2.4)	5.0^c^ (3.3–7.6)
Model 2	0.9 (0.5–1.3)	1.0	3.2^c^ (2.2–4.6)	5.4^c^ (3.3–8.7)
**WOMEN**				
** Hypertension**				
Model 0	0.7^a^ (0.5–1.0)	1.0	2.5^c^ (1.9–3.3)	4.7^c^ (3.4–6.4)
Model 1	0.8 (0.6–1.1)	1.0	2.2^c^ (1.7–2.9)	3.5^c^ (2.5–4.8)
Model 2	0.9 (0.6–1.5)	1.0	2.1^c^ (1.4–3.3)	3.4^c^ (2.0–6.0)
** Diabetes mellitus**				
Model 0	0.4^a^ (0.2–0.8)	1.0	2.4^b^ (1.4–4.1)	5.1^c^ (2.9–8.9)
Model 1	0.4^a^ (0.2–0.9)	1.0	2.0^a^ (1.1–3.4)	3.3^c^ (1.8–5.9)
Model 2	0.4 (0.1–1.8)	1.0	1.4 (0.5–3.9)	2.8 (0.9–8.4)
** Hypercholesterolemia**				
Model 0	0.6^c^ (0.5–0.7)	1.0	2.1^c^ (1.7–2.6)	2.8^c^ (2.2–3.7)
Model 1	0.6^c^ (0.5–0.8)	1.0	1.9^c^ (1.5–2.3)	2.1^c^ (1.6–2.8)
Model 2	0.7^a^ (0.5–1.0)	1.0	1.9^c^ (1.4–2.7)	1.9^b^ (1.2–3.1)
**BOTH SEXES**				
** Hypertension**				
Model 0	0.7^b^ (0.5–0.8)	1.0	2.3^c^ (1.9–2.9)	4.6^c^ (3.6–5.9)
Model 1	0.7^b^ (0.5–0.9)	1.0	2.1^c^ (1.7–2.6)	3.5^c^ (2.7–4.6)
Model 2	0.7^b^ (0.6–0.9)	1.0	2.1^c^ (1.7–2.5)	3.5^c^ (2.7–4.6)
** Diabetes mellitus**				
Model 0	0.4^a^ (0.2–0.8)	1.0	3.1^c^ (2.0–4.8)	5.3^c^ (3.3–8.7)
Model 1	0.4^a^ (0.2–0.8)	1.0	2.4^c^ (1.5–3.8)	3.2^c^ (1.9–5.3)
Model 2	0.4^a^ (0.2–1.8)	1.0	2.3^c^ (1.5–3.7)	3.1^c^ (1.9–5.3)
** Hypercholesterolemia**				
Model 0	0.7^c^ (0.6–0.8)	1.0	2.6^c^ (2.2–3.1)	3.8^c^ (3.1–4.8)
Model 1	0.7^c^ (0.6–0.8)	1.0	2.2^c^ (1.8–2.6)	2.6^c^ (2.1–3.3)
Model 2	0.7^c^ (0.6–0.8)	1.0	2.2^c^ (1.8–2.6)	2.6^b^ (2.1–3.3)

a *p*-value <0.05;

b *p*-value <0.01;

c *p*-value <0.001.

Model 0: Non Adjusted.

Model 1: Adjusted for age, sex, residence,

Model 2: Adjusted for age, sex, residence, physical activities, cigarette smoking, alcohol drinking, fruit and/or vegetable consumption, using lard/suet.

**Figure 3:**
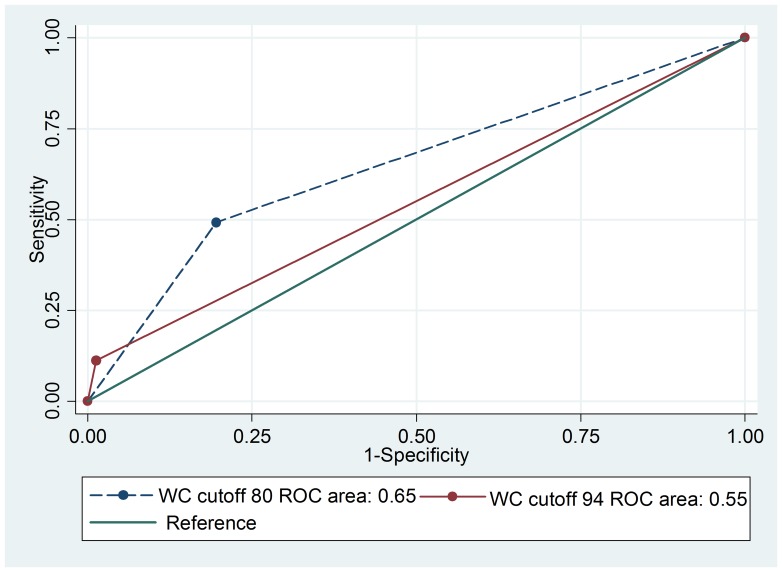
ROC curve comparing two WC categories for predicting hypertension, diabetes mellitus and hypercholesterolemia in men.

For men, the areas under the ROC curve comparing a WC cutoff of 80 cm and 94 cm (the cutoff recommended by WHO for men) were 0.65 and 0.55, respectively, with *p*-value <0.001 (Figure 3). The sensitivities for detecting hypertension or diabetes mellitus or hypercholesterolemia using a WC cut-off of 80 cm and 94 cm were 49.2% and 11.2% respectively, and the corresponding specificities were 80.5% and 98.7%, respectively.

## Discussion

To the best of our knowledge, this is the first study using nationwide data to examine the appropriate BMI and WC cutoff for overweight and central obesity among adults in Cambodia. In general, the significant association of subjects with hypertension and hypercholesterolemia was found in those with BMI ≥23.0 kg/m^2^ and with WC >80.0 cm in both sexes in all three models. The AUC was significantly greater in both sexes when BMI of 23.0 kg/m^2^ was used as the cutoff point for overweight compared to that using the WHO BMI classification for overweight (BMI ≥25.0 kg/m^2^) for detecting the three cardiovascular risk factors. Similarly, the AUC was also higher in men when WC of 80.0 cm was used as cutoff point for central obesity compared to that recommended by WHO (WC ≥94.0 cm in men). Based on these results, the prevalence of overweight was almost doubled, from 13.5% to 25.5%. The prevalence of central obesity was also significantly augmented from 11.8% in men and 16.9% in women to 20.0% in both sexes.

These findings are similar to results in some studies in Asian populations [1], [4], [6], [9]. The associations were independent of the effects of potential confounders such as age, sex, residence, physical activities, cigarette smoking, alcohol drinking, fruit or vegetable consumption, and using lard or suet as cooking oil. The risks of developing metabolic syndrome increased significantly in accordance with the increase of BMI and WC. These findings are also consistent with those found in other studies in Asia [22]–[25].

The risk of hypertension for both sexes was similar at a given BMI and WC levels. However, the risk of diabetes mellitus and hypercholesterolemia was higher in men than in women at the same BMI levels. This result suggests that men are more likely to develop diabetes and hypertension than women at a lower BMI and WC levels. This result is consistent with findings in other studies which found that men tend to develop metabolic syndrome at earlier ages than women [21], [26], [27].

Significant associations between WC and hypertension, diabetes mellitus, and hypercholesterolemia were generally seen among those with WC >80.0 cm in both sexes in all three models. Therefore, WC of 80 cm appears to be an appropriate cutoff point to define central obesity for adults aged 25–64 years in Cambodia. This finding is similar to findings in other studies which aimed to determine an appropriate cut-off of WC for Asians [6], [28]. After including potential risk factors into the models (model 1 and 2), risk of diabetes among men with WC >90.0 cm were approximately two fold higher than that in the reference group (WC >70.0–80.0 cm), but this association was not statistically significant. In women, diabetes mellitus was not significantly associated with those with WC >80.0 cm in model 2. It is observed that men with WC >90.0 cm had more than two times the risk of hypercholesterolemia than women with the same WC size.

The strengths of this study include the large nationally representative samples. Moreover, research methodology used in this study was adapted from WHO STEPwise approach to surveillance (STEPS) which is a simple, standardized method for collecting, analyzing, and disseminating data in WHO member countries [15]. In addition, blood pressure, blood glucose, cholesterol, and other anthropometric variables used in this study were based on real measurements, not based on self-report. However, several limitations should also be considered. Originally, data were collected from 5,433 subjects. In our analyses, 418 participants were excluded because of some missing or abnormal values. Thus only 5,015 participants were included in our analyses. This might affect the validity of the study. Additionally, data from a cross-sectional design was used in this study. Thus a causal relationship cannot be definitively established. The final limitation of this study concerns self-reported measures for many variables which may lead to over or under ascertainment of the true measurement.

In conclusion, the increased risk of hypertension, diabetes mellitus, and hypercholesterolemia was statistically significant in both sexes with a BMI of ≥23.0 kg/m^2^ and WC of >80.0 cm. As the definition of the cutoff value for “normal” BMI and WC in a population should depend on identifying the risk association with non-communicable diseases, our findings may be used to derive the normal cutoff values of BMI and WC. Therefore, we suggest that a BMI cutoff of 23.0 kg/m^2^ and WC cutoff of 80.0 cm may be appropriate for the designation of over-weight and central obesity in adult men and women in Cambodia. Our findings are useful for policy makers as well as for the Ministry of Health of Cambodia for development of strategies to prevent NCDs which are gradually emerging in Cambodia.
